# Task-induced hyperfunction of the left dorsolateral prefrontal cortex in patients with primary insomnia: a near-infrared spectroscopy study during a verbal fluency task

**DOI:** 10.3389/fpsyt.2026.1730858

**Published:** 2026-02-06

**Authors:** Ting Wu, Hong He, Bo Yu, Qiang Zhang, Shaohui Li, Yonghong Wu, Mingjin Huang

**Affiliations:** 1 The Third Hospital of Mianyang, Sichuan Mental Health Center, Mianyang, China; 2 North Sichuan Medical College, Nanchong, China

**Keywords:** functional near-infrared spectroscopy, hyperfunction, prefrontal cortex, primary insomnia, verbal fluency task

## Abstract

**Introduction:**

In this study, we aimed to use functional near-infrared spectroscopy (fNIRS) to investigate the differences in the functional brain activation of patients with primary insomnia (PI) and that of good sleepers (GSs) without other comorbidities or structural abnormalities during a verbal fluency task (VFT).

**Methods:**

Thirty PI participants and 30 GSs completed a clinical questionnaire and performed VFT during an fNIRS scan. A two-sample t-test was performed to compare brain activation on a cognitive test between patients with PI and GSs.

**Results:**

We observed higher brain activation in the left dorsolateral prefrontal cortex in the insomnia group during VFT compared to GSs (p < 0.05).

**Discussion:**

Our findings may contribute to an understanding of the neural mechanisms in patients with PI.

## 1 Introduction

Insomnia is a common health complaint characterized by poor sleep quantity and quality coupled with impaired cognitive performance and daytime functioning (including fatigue, exhaustion, reduced alertness, dysphoria, and other symptoms). Primary insomnia (PI) is among the most prevalent chronic sleep disorders that cannot be attributed to any medical, psychiatric, or environmental factors, affecting up to 10% of the adult population.

Insomnia is often closely associated with hyperarousal and increased cortical activation. The hyperarousal theory, a robust framework in insomnia research, posits the interaction of psychological and physiological factors expressed in terms of somatic, cognitive and cortical activation, with evidence from multiple areas including neuroimaging, neuroendocrinology and electrophysiology. (1–3)Increased cognitive activity probably occurs as a result of cortical arousal. Hence, insomniacs may experience physiologic hyperarousal in both central cortical and peripheral autonomic nervous systems. A previous study showed that primary insomnia was associated with impaired neuropsychological performance (4). However, most studies found that patients with insomnia experience minor differences compared with good sleepers (GSs) in objective cognitive performance. The possible explanation for the subtle cognitive deficits might be the compensatory effort. Therefore, cognitive neuroimaging techniques can elucidate subtle alterations in cognitive processes, which may not be readily apparent through traditional behavioral performance measures. Regions exhibiting aberrant coactivation may provide critical insights into the underlying neural mechanisms of the insomnia-associated daytime dysfunction. According to early neuroimaging findings in PI, increased function during sleep is linked to abnormal physiological arousal in several key brain regions, including the ascending reticular activating system, hypothalamus, thalamus, basal forebrain, and ventromedial prefrontal cortex (5, 6). Although preliminary neuroimaging studies suggested that higher excitability in the bilateral dorsolateral prefrontal cortex (DLPFC) in patients with insomnia compared with healthy controls, the understanding of the basic neural mechanisms of sleep disorders remains unclear. Moreover, the results of cognitive performance and alterations in different brain regions when patients with insomnia execute different cognitive tasks are equivocal. For example, significant changes in task-related brain activation or behavioral performance during the Stroop task were not observed between patients with insomnia and GSs (7). However, another study using functional magnetic resonance imaging (fMRI) combined with the N-back working memory task found that PI patients showed reduced activation of task-related working memory regions and no difference in cognitive performance compared with healthy subjects (8). Additionally, the first resting-state network study using fMRI and electroencephalography (EEG) suggested that the increased coactivation of the insula was associated with insomnia (9). Thus, the exact mechanism remains poorly understood, integrating traditional psychological models of insomnia with contemporary neurobiological evidence will enhance our comprehension of insomnia-associated cognitive impairments. Functional near-infrared spectroscopy (fNIRS) has been extensively employed to observe brain activity patterns in individuals with neurological and psychiatric disorder().The VFT task is a widely utilized assessment tool for verbal and executive control abilities, which are connected to basic neurocognitive functions like working memory, motivation, and attention (10). Regarding alterations relevant to insomnia, neuroimaging studies (11–13) based on functional near-infrared spectroscopy (fNIRS) have recently reported regional brain activity changes in insomnia compared to healthy sleepers.

Thus, this preliminary study sought to investigate the subclinical changes in the verbal fluency task-related brain activity of PI by using functional NIRS and further explore whether this effect was associated with behavioral performance. Accordingly, we hypothesized that patients with PI would show increased activation while performing a verbal fluency task (VFT).

## 2 Materials and methods

### 2.1 Patients

Thirty PI patients were recruited from the Psychiatry Department of the Third Hospital of Mianyang. PI was diagnosed using the diagnostic criteria for PI according to the Diagnostic and Statistical Manual of Mental Disorders-fifth Edition (DSM-5) criteria under a structured clinical interview conducted by two experienced psychiatrists. Additionally, 30 age- and sex-matched GSs were enlisted through WEIXIN advertisements.

Eligible subjects were asked to complete the Pittsburgh Sleep Quality Index (PSQI) and insomnia severity index (ISI) as additional measures of self-reported sleep quality and insomnia severity, while anxiety and depression levels were measured using the Hamilton Anxiety Rating Scale (HAMA) and Hamilton Depression Rating Scale (HAMD24).

Exclusion criteria included (1) any past or present DSM-5 Axis I disorder; (2)HAMA score > 14, HAMD score > 17;(3)sleep disorder other than insomnia (e.g., obstructive sleep apnea [OSA] or restless legs syndrome); (4) a history of serious medical or neurological disease; (5) intake of any prescription psychotropic or hypnotic medication in the last 2 weeks, (6) shift workers or pregnant women. All GSs had an average sleep efficiency of ≥90% which was estimated based on PSQI (14), and they had no sleep complaint.

The study protocol was approved by the Ethics Committee of The Third Hospital of Mianyang. All individuals involved in the study were fully informed about the study and provided written informed consent.

### 2.2 Verbal fluency task

The VFT comprised a 30-s pre-task resting phase, a 60-s task period, and a 60-s post-task resting phase. During the pre- and post-task resting periods, the participants repeated counting from 1-2-3-4-5. During the task period, the participants constructed as many phrases as possible using three commonly used Chinese characters, such as 白 (white), 蓝 (blue), and 火 (fire). Every character during the task period was changed every 20 s. All participants were instructed to refrain from consuming coffee or tea for at least 24 h before the experiment. The total number of phrases articulated by each individual was recorded.

### 2.3 fNIRS acquisition

**Figure 1:**
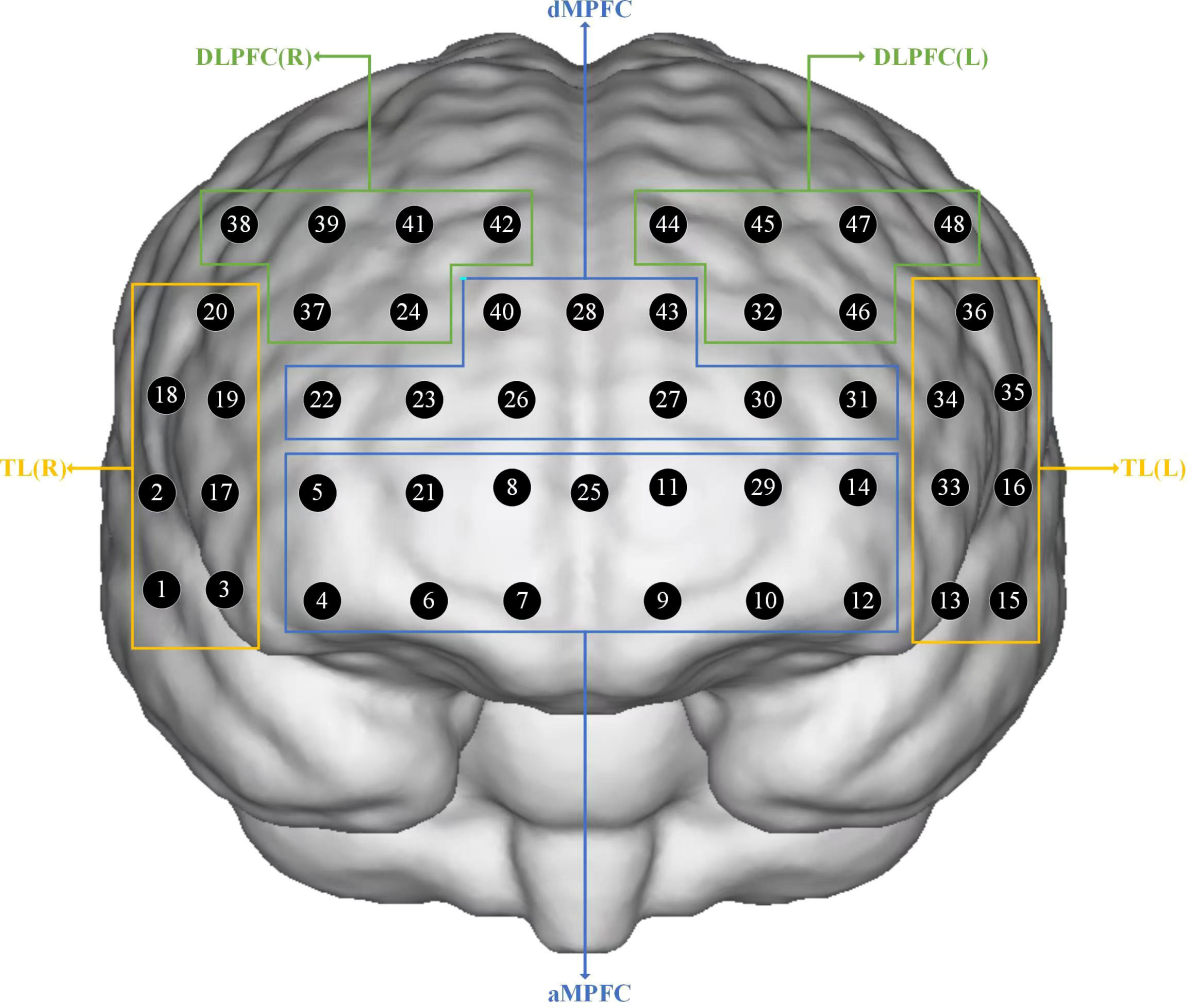
Schematic of arrangement of fNIRS channels. DLPFC, dorsolateral prefrontal cortex; aMPFC, anterior medial prefrontal cortex; dMPFC, dorsolateral medial prefrontal cortex; TL, temporal lobe; R, right; L, left.

A 48-channel near-infrared optical imaging system (NirScan-9000DT, Danyang Huichuang Medical Equipment Co, Ltd, China), which operated at three wavelengths (730/808/850 nm) with the sampling set at 11 HZ, was used to measure the relative changes in oxygenated hemoglobin (oxy-Hb). We utilized a probe set containing 15 light sources and 16 light detectors, which was arranged in the dorsolateral prefrontal and temporal cortices of 10/20 electrodes. We mapped the 48 channels to the corresponding areas of a brain model. These channels were categorized as the right and left dorsolateral prefrontal cortex (DLPFC), anterior medial prefrontal cortex (aMPFC), dorsal medial prefrontal cortex (dMPFC), and right and left temporal lobes (TLs), which constituted six regions of interest (ROIs). **Figure 1** presents the channels and the corresponding brain regions.

### 2.4 Data processing and analysis

fNIRS data were processed using the NirSpark analyzing software package as following steps. (1) the unsatisfactory time intervals with sudden, obvious, discontinuous noise were excluded;(2) the artifacts induced by motion and environment were corrected (the standard deviation of threshold =6.0; the amplitude of threshold=0.5); (3)a band-pass filter (0.01-0.2HZ)) was applied to remove the slow drift of physiology noise and environmental noise. (4) the raw optical density values were converted into concentration changes in oxygenated hemoglobin (oxy-Hb) through the modified Beer Lambert Law (15).(5)We calculated the intertrial mean of differences between the oxy-Hb concentration changes during the target period in each channel;(6)the regional value of the difference between oxy-Hb changes in the target period and baseline was extracted by averaging categorized channels based on the specified ROI. Subjects having the greater than 3 blocks or/and 10 channels eliminated due to poor signal quality were removed.

All statistical analyses were completed using IBM SPSS version 25 (IBM Corporation, Armonk, NY, USA). The statistical threshold was set at p < 0.05. An independent two-sample two-tailed t-test was used to compare variables between the groups. For variables that violated the normality assumption, the non-parametric Mann–Whitney U test was employed. The statistical results were corrected for multiple comparisons across channels by the false discovery rate (FDR, 16). The normality of the data was evaluated via the Shapiro-Wilk test. We conducted correlation analysis using Pearson’s method to examine the association between behavioral performance and fNIRS data.

## 3 Results

### 3.1 Behavioral results

**Table 1** presents social demographic data. The groups were equivalent in age and gender, which were within normal range values. As expected, individuals with insomnia presented significantly higher scores on PSQI (t = 26.7, p < 0.00) compared with GSs. Given the Insomnia Severity Index (ISI) scores violated the normality assumption in the insomnia group (p < 0.05), the nonparametric Mann-Whitney U test was employed, which confirmed significantly higher scores in patients compared to good sleepers (U = 105, p < 0.001). However, no significant differences in the number of words on the VFT were observed for PI patients and controls (t = −1.33, p = 0.19).

**Table 1:** Demographic and clinical characteristics of participants.

Characteristic	Patients with PI	Good sleepers	t-values	P-values
	Mean (SD)	Mean (SD)		
Gender (M/F)	10/20	14/16		0.29
Age(Y)	48.97 ± 7.92	45.07 ± 10.48	1.63	0.11
Duration(Y)	3.2 ± 1.3	/	/	/
PSQI	16.27 ± 2.60	2.63 ± 1.03	26.7	<0.001***
ISI	19.7 ± 4.90	1.07 ± 1.31	20.12	<0.001***
Number of phrases	9.33 ± 1.97	9.96 ± 1.71	-1.33	0.19

PI, primary insomnia disorder; PSQI, the Pittsburgh Sleep Quality Index; ISI, Insomnia Severity Index; ***p < 0.001.

### 3.2 Brain activity during VFT

**Figure 2:**
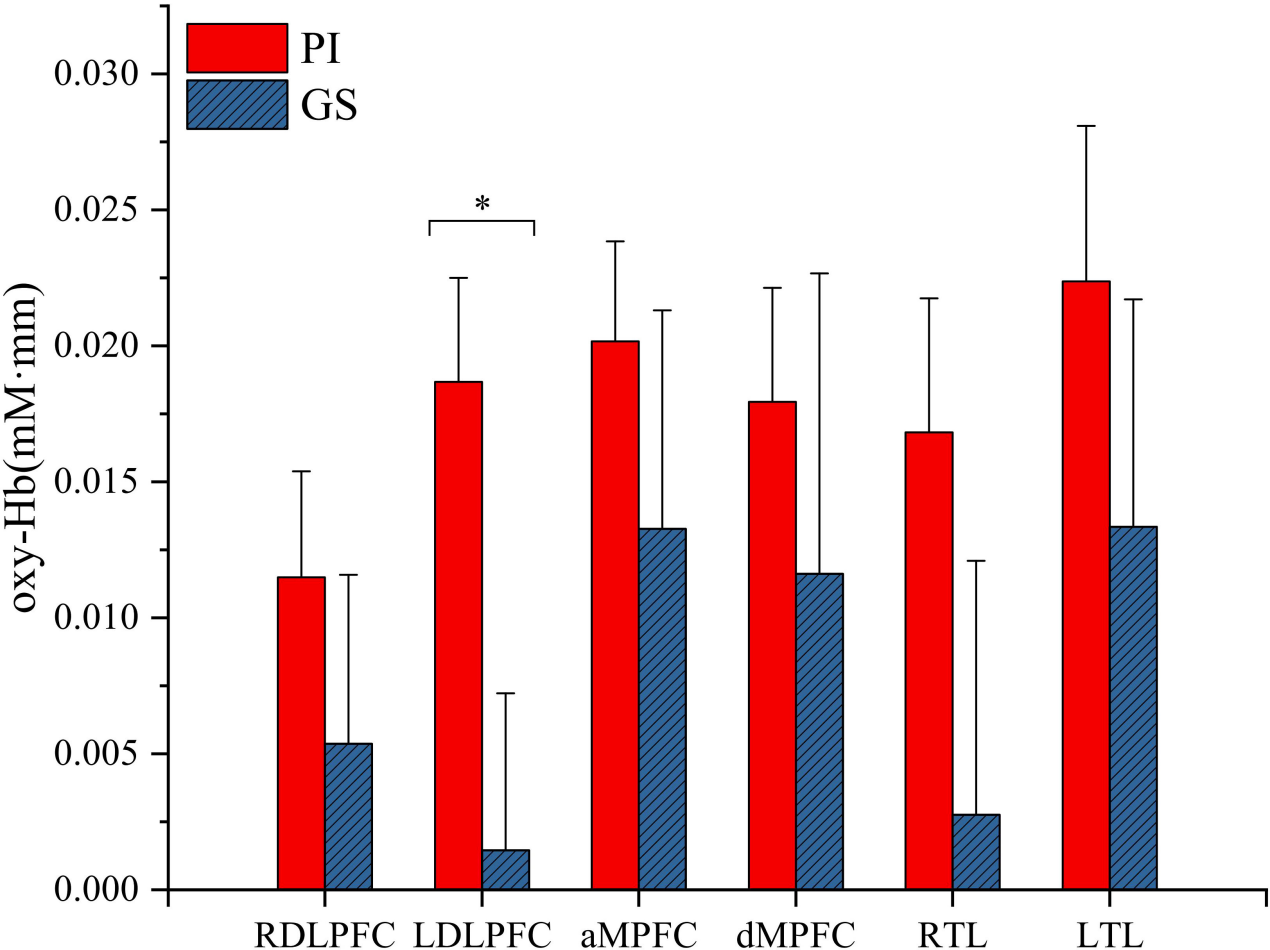
Hemodynamic changes during performance in the VFT task. RDLPFC, right dorsolateral prefrontal cortex; LDLPFC, left dorsolateral prefrontal cortex; aMPFC, anterior medial prefrontal cortex; dMPFC, dorsolateral medial prefrontal cortex; RTL, right temporal lobe; LTL, left temporal lobe; *p < 0.05.

Compared with the GS group, patients with PI showed significantly higher task-related oxy-Hb changes in the left DLPFC (t = 2.49, p < 0.05; **Figure 2**). However, there were no significant differences in oxy-Hb signals in the regions of right DLPFC (t = 0.835, p = 0.407), MPFC (anterior MPFC t = 0.78, p = 0.439; dorsal MPFC t = 0.535, p = 0.594), and TL (right TL t = 1.333, p = 0.188; left TL t = 0.89, p = 0.377) activated by the VFT task between the PI and GS groups.

### 3.3 Correlation between oxy-Hb values in abnormal regions and PSQI and ISI scores

No significant correlation between oxy-Hb values in the left DLPFC and PSQI (R = 0.16; p = 0.39) and ISI (R = 0.17; p = 0.38) scores was identified.

## 4 Discussion

The present study investigated the neural activation during the VFT in patients with PI. Patients with PI showed higher brain activation in the left DLPFC during VFT compared with the patients in the GS group. This finding suggests task- and region-specific hyperactivation in PI patients within the specific context of the verbal fluency task. The left prefrontal regions are associated with language and executive control, and the VFT task consistently activates the left hemisphere (17). However, we did not observe a significant correlation between oxy-Hb values in these regions and clinical symptoms (PSQI and ISI scores), suggesting that the observed hyperactivation may be task-specific rather than directly tied to global symptom severity. Potential explanations include: (1) oxy-Hb may reflect neural activity in specific cortical regions not captured by subjective scales, or (2) the subjective nature of the scales introduces variability unrelated to physiological changes. Thus, future studies should combine multimodal assessments to clarify this discrepancy.

Previous neuroimaging studies reported increased brain activity in insomnia compared with healthy controls under specific task conditions or during a resting state. Our results align with these studies in demonstrating content-dependent hyperactivation. For example, PI patients exhibit increased spontaneous activity and connectivity during daytime, which may contribute to difficulty falling asleep (18, 19). Wang et al. (20) and Chen et al. (9) reported that insula activity was significantly increased in PI patients, suggesting that the aberrant activity in the left insula may be linked to sleep-related anxiety. Earlier structural studies demonstrated that patients with insomnia have reduced gray matter volume in several brain areas (e.g., the left prefrontal, temporal, and anterior cingulate cortex) (5, 21, 22). These structural changes and task-evoked hyperactivation in patients with insomnia may reflect the impaired deactivation of executive networks. Specifically, the observed left- DLPFC hyperactivation reflects the task-relevant neural response pattern inherent to the VFT paradigm, which is known to robustly engage this region for phonemic retrieval and verbal executive control. This provides a focused context for interpreting its significance in insomnia. This finding leads us to hypothesize that the left DLPFC hyperactivation during a verbal task may reflect an extended state of cognitive hyperarousal and impaired executive disengagement. This interpretation, while speculative and derived from our specific task and modality, is aligns with the clinical phenotype of insomnia, where patients struggle to disengage from verbal rumination and goal-directed cognition at sleep onset—processes heavily reliant on left-lateralized prefrontal networks. In our study, patients with insomnia showed no difference in behavioral performance on the VFT task compared with the GS group, although daytime dysfunction is a common complaint in these patients. Given the hyperactivation in the left DLPFC in the absence of cognitive impairment, we assume that compensatory neural activation may serve as a mechanism to mitigate potential underlying cognitive deficits. Collectively, our research results provide preliminary, task-specific evidence for altered regional activity in PI patients, which requires further validation.

However, the literature on task-related brain activation in insomnia provides inconsistent findings. Spiegelhalder and Hwang demonstrated no significant difference in brain activation in patients with insomnia during the Stroop task (7, 23). Li et al. (24) observed lower activation in the left hemisphere during a spatial working memory task. More interestingly, compared with healthy controls. Interestingly, PI patients showed heightened activity in response to emotional (25) or stressful stimuli (26). Additionally, discrepancies also exist for the same cognitive task: Son and colleagues used fMRI with the n-back task to investigate that working memory-related prefrontal brain activation showed increased cerebral responses in the right lateral inferior prefrontal cortex and right superior temporal pole in patients with insomnia compared to healthy controls (27), while another study reported reduced prefrontal activation in patients with insomnia and minor performance defects in the n-back task (8). Our findings also contrast with a recent fNIRS study using the VFT, which reported hypoactivity of oxy-Hb concentrations in chronic insomnia (11, 28). However, Sun et al., observed oxy-Hb changes in the left prefrontal cortex of some patients with chronic insomnia tend to increase progressively with decreasing sleep quality.

These inconsistencies mya arise from: (i) each cognitive test was designed to measure distinct targets within a range of constructs in the cognitive process, and some discrepancies may arise from variations in task difficulty; (ii) the differences in insomnia severity across studies; (iii) some participants with insomnia were taking hypnotic medications during the research, which primarily target the histaminergic arousal system. Future studies should systematically evaluate whether left DLPFC hyperfunction generalizes to other tasks or brain regions in PI patients. Additionally, stratification by gender, age, symptom severity, and treatment history is needed. Importantly, integrating resting-state functional connectivity measures in future work could help elucidate how state-dependent left DLPFC hyperactivation during tasks relates to trait-like alterations in intrinsic connectivity within and between networks such as the default mode network (DMN). This system approach will provide a more comprehensive understanding of whether hyperactivity represents a network dysregulation.

The frontal lobe, particularly the left DLPFC, is critical for executive functions, emotional regulation, and memory retrieval. Overactivation in frontal and temporal regions is linked to emotional dysregulation, such as anxiety and depression. Furthermore, insomnia frequently co-occurs with major depressive disorder (MDD). Xu et al. (29) found that MDD patients with insomnia exhibited higher excitability in the left prefrontal cortex, which was negatively correlated with sleep quality. Similarly, Dai et al. (30) proposed compensatory hyperactivation as a mechanism for insomnia in MDD.

Abnormalities in the bilateral DLPFC are implicated in insomnia (31). Consequently, both the left and right DLPFC emerged as prominent targets for non-invasive neurostimulation techniques, such as repetitive transcranial magnetic stimulation (rTMS), in the treatment of insomnia. The normalization of DLPFC hyperactivity after treatment (32, 33) further supports its role. In addition,1 Hz rTMS over the left dorsal lateral prefrontal cortex (DLPFC) has been widely used in the treatment of insomnia disorders, and subjective sleep improvements are commonly reported (34, 35). However, the mechanisms underlying the effect of 1 Hz rTMS administration on the left DLPFC in patients with chronic insomnia are not exactly clear.Our observation of hyperactivity in the left DLPFC in insomnia patients may contribute to understanding this mechanism. Greater understanding of the pathophysiology of insomnia may provide important information regarding how and under what conditions the disorder develops, as well as potential targets for prevention and treatment.

### 4.1 Limitations

The current study has several limitations that should be addressed in future investigations. First, our findings are specific only to the VFT and the left DLPFC. Future work should assess whether hyperactivation extends to other tasks or regions, including resting-state networks. Second, prior studies suggested sex-specific neural responses in PI (e.g., 19), but our small sample precluded subgroup analysis. Hence, our results in chronic insomnia must be considered preliminary and replication in larger, stratified cohorts is needed. Third, chronotype, insomnia episode duration, and medication history were not assessed. Additional objective sleep measurements (such as PSG), detailed illness course, and medication records should be considered in future studies. Fourth, fNIRS can only measure Hb concentration changes in upper cortical areas, whereas deeper structures (e.g., the insula) require multimodal imaging. Hence, combining fNIRS with complementary imaging techniques such as EEG and MRI would improve both the temporal and spatial resolution of our investigations.

### 4.2 Conclusion

Our study highlights left DLPFC hyperactivation during the VFT in PI patients. Which may potentially reflecting a compensatory neural response within this specific paradigm. However, the heterogeneity in pathophysiology of insomnia demands further research with standardized task paradigms and stratified participant cohorts.

## Data Availability

The original contributions presented in the study are included in the article/supplementary material. Further inquiries can be directed to the corresponding author.
